# Application of Nanotechnology in Orthodontic Materials: A State-of-the-Art Review

**DOI:** 10.3390/dj8040126

**Published:** 2020-11-09

**Authors:** Alberto De Stefani, Giovanni Bruno, Giorgia Preo, Antonio Gracco

**Affiliations:** Faculty of Dentistry, University of Padova, via Giustiniani 2, 35100 Padova, Italy; giobruno93@gmail.com (G.B.); giorgiapreo@gmail.com (G.P.); antoniogracco@gmail.com (A.G.)

**Keywords:** nanotechnology, nano-dentistry, orthodontics, nanomedicine, nanomaterials in dentistry, nanobiomaterials, nanorobots in dentistry, silver nanoparticles, nanocomposite, dentifrobots, nanorobots, fullerene nanoparticles

## Abstract

Nanotechnology refers to the science that manipulates matter at molecular and atomic levels, and studies matter at the nanoscale level to detect and exploit the useful properties that derive from these dimensions; materials with components less than 100 nm in at least one dimension are called nanomaterials. Nanotechnology is applied in many fields, such as medicine (nanomedicine) and dentistry (nano-dentistry). The purpose of these innovations and research in this field is to improve human life and health. This article aims to summarize and describe what the most recent and known innovations of nanotechnology in dentistry are, focusing on and paying particular attention to the branch that is orthodontics, and on the application of new nanomaterials in the realization, for example, of orthodontic elastomeric ligatures, orthodontic power chains, and orthodontic miniscrews. We also address a very important topic in orthodontics, which is how to reduce the friction force.

## 1. Introduction

Nanotechnology is the science that manipulates matter at molecular and atomic levels. Different fields of application have been considered by researchers to detect and exploit the useful properties that derive from components at the nanoscale level. The term was coined by N. Taniguchi in 1974 at the Tokyo Science University [1].

Materials with components smaller than 100 nm in at least one dimension are called nanomaterials. These components may include grains, fibers, clusters, nanoholes or a combination of these forms. In one dimension are defined sheets; in two dimensions, nanowires and nanotubes; and in three dimensions, they are called quantum dots. A fundamental aspect of these new particles is that they increase the surface area per unit mass compared to bigger particles, significantly modifying the physical and chemical properties of the material [2]; the novel mechanical properties of nanomaterials are the object of this review.

Nanotechnologies are applied in many fields, including medicine (nanomedicine) and dentistry (nano-dentistry). Nanomedicine is the science of preventing, diagnosing and treating disease and preserving and improving human health using nanosized particles [3]. The purpose of these innovations and research in this field is to improve human life and health.

The toxicity of nanomaterials and nanoparticles is a hotly debated topic: some studies that we have reviewed have not addressed it; others underline the fact that today, there is still little evidence about it, especially in the dental sector. Nanotechnology can make the clinic simpler and easier, but some authors point out that there are currently limitations relating to the topic of safety [4].

Nanomaterials have particular characteristics: the surface area/volume ratio (volume-specific surface area) of nanoparticles is greatly increased, and therefore, they are much more reactive compared with larger particles with the same composition. They have a high absorption rate in the body, in particular, through the skin, digestive tract and lungs; after prolonged use, they can accumulate in various organs, such as the lungs and digestive tract. They have the potential to cause DNA mutations, cell damage and inflammation. The relevance of these findings in dentistry is unknown [4,5]. In dentistry, nanoparticles are intentionally incorporated into products to improve their qualities. The materials that intentionally release these molecules are few and used in areas other than orthodontics (for example, for sprays for intraoral scans or CAD/CAM design and manufacture). It is estimated that nanoparticles are present in approximately 3500 dental materials [5].

All these factors make it clear that nanotechnology is a relatively new field and still unexplored in some aspects. Many ideas are still difficult to implement due to technological, biological and ethical challenges. To date, further research and studies are needed for the application of this field as regards dentistry [4].

A new branch called nano-dentistry has become more famous in recent years, and it involves the effort of different researchers and clinicians for the development of new materials. Nano-dentistry can be described as the science that uses nanostructured materials and technologies to diagnose, treat and prevent oral and dental disease [2]. A common interest is to improve patients’ oral health, decreasing the invasiveness of treatments and increasing compliance with doctors [6].

Conservative, endodontic, anesthesiology, aesthetics and orthodontics are the branches mainly involved. In recent years, much attention has been paid to dentistry nanorobots and tissue engineering, but also to studies regarding stem cells for bone augmentation and cartilage regeneration [7,8,9,10].

However, some applications of nanotechnology in dentistry have already been tested and are now used in various sectors; others need further studies and scientific research [11].

This article aims to summarize and describe what the most recent innovations of nanotechnology in dentistry are; the authors took care in reviewing the most recent literature, to evaluate and report, in this research, the most significant innovations in the orthodontic field, comparing different articles and literature reviews and focusing the attention on the application of new nanomaterials in the realization, for example, of orthodontic elastomeric ligatures, orthodontic power chains and orthodontic miniscrews. We also address a very important topic in orthodontics, which is how to reduce the friction force.

To summarize, the following paragraphs are the different fields of application of nanotechnology in orthodontics on which clinicians and researchers are focusing their efforts (Table 1).

## 2. Methods

The authors reviewed the available literature and selected articles using the PubMed search engine. The following keywords were entered: nanotechnology; nano-dentistry; orthodontics; nanomedicine; nanomaterials in dentistry; nanobiomaterials; nanorobots in dentistry; silver nanoparticles; nanocomposite; dentifrobots; nanorobots; fullerene nanoparticles. The most complete and recent articles on this scientific topic were searched from the last 15 years. Thanks to the abstracts of the articles, those that focused their attention in particular on dentistry and orthodontics were then sought. The authors identified and selected the items to include; they researched the most investigated topics in the literature regarding nanomaterials and nanotechnology in orthodontics, and then organized the articles into macro-topics and subsequently evaluated them with the aim of collecting the most useful and scientific information from each. In this way, the materials that today have a significant role for dentists were selected. The authors’ aim was to pay particular attention to orthodontics, and they therefore divided this article into sections dedicated to the most used materials in this field.

## 3. Orthodontic Elastomeric Ligatures

Orthodontic elastomeric ligatures (OEM) are synthetic elastic modules of polyurethane material, with advantages such as the quickness of application and patient’s comfort; they are often a wise solution in clinical practice because they are inexpensive.

During orthodontic therapy, there is an inevitable microbiological colonization of the material, firstly, due to the obvious increase in the retention surface: in fact, there is an increase in the accumulation of bacterial plaque, bacterial colonization and enamel decalcification, with a worsening of bleeding rates. The increase in bacterial plaque is also partially related to the irregular surfaces of the orthodontic auxiliaries. For these reasons, maintaining correct oral hygiene and self-cleansing are indeed more difficult. Bacterial counts increase approximately thirty times at six weeks with an increase in *Streptococcus mutans*, *Staphylococcus aureus* and *Lactobacilli*.

Several studies have tried to highlight the associations based on the chemical characteristics of different elastic ligatures and the requirements of modifications for hampering the microbiological biofilm’s effects on oral health. Some studies have evaluated ligatures that release fluoride, but the results are varied and controversial: some highlight how, in particular, there is long-term ineffectiveness linked to the inability of the ligatures to guarantee a prolonged release over time; they also seem to be ineffective even against the decalcification [12,23,24].

It has been proposed that elastomeric ligatures can act as a support for the transport of nanoparticles, which can be molecules with anticariogenic or anti-inflammatory characteristics and/or antibiotic drugs (such as benzocaine) incorporated into the elastomeric matrix. Additionally, medicated wax applied to orthodontic brackets that reduces the pain associated with mucosal irritation caused by the brackets by slowly and continuously releasing benzocaine was shown to be significantly more effective [25].

The most recent studies seem to evaluate the potentiality of the association of ligatures with silver nanoparticles, a material that appears to have the ability to counter dental biofilm and decrease the enamel demineralization caused by the accumulation of bacterial plaque, without affecting the mechanical characteristics of the material itself and therefore the effectiveness of orthodontic therapy. Nanotechnology in dentistry applied to materials is innovative for the concept of leading to better anticariogenic characteristics; the silver particles have been incorporated into different materials and can be obtained with minimally invasive methods from elements such as flowers and mushrooms. It is known that silver has a superior antibacterial property compared with other metals; it has a strong cytotoxic effect on a wide range of microorganisms: the mechanism is not very clear; it probably acts by denaturing the enzymes of the respiratory cycle and DNA synthesis. Furthermore, silver is not very toxic and has good compatibility with human cells. Today, however, other studies are needed to determine their actual biocompatibility [12].

## 4. Orthodontic Power Chains

Power chains have been used daily in every orthodontic practice since their development in the late nineteen sixties. They are generally composed of polymeric materials (polyesters or polyethers) formed through a process of polymerization. They present different clinical advantages: they are affordable, user-friendly and easy to adjust to every patient and provide light and continuous forces. They are characterized by high flexibility, and they enhance space closure in extraction cases. On the other hand, power chains also have unfavorable characteristics: it is amply demonstrated that their mechanical effectiveness is limited in time, and, for this reason, they must be replaced periodically. Both intrinsic and external factors affect the strength of orthodontic power chains and determine permanent deformation. The intrinsic factors are the material composition, production methods and external morphology. The external factors are the oral cavity temperature, pH and moisture absorption. They also have hydrophilic characteristics and undergo discoloration over time; they absorb fluids from the oral cavity and are therefore not very appropriate for maintaining oral health [13].

In a study conducted in Taiwan by Cheng et al., the authors tried to improve the physical properties of power chains by performing a surface treatment called nanoimprinting. The treatment consists of creating nanostructures on the surface of the chains called nanopillars. The results seem encouraging, as this treatment converts the material from hydrophilic to hydrophobic and alleviates the shortcomings of these orthodontic auxiliaries [13].

## 5. Orthodontic Bands

Fixed orthodontic treatment often requires the insertion of dental bands, which are often essential for orthodontic movements. However, these auxiliaries can cause the retention of bacterial plaque, especially in the posterior dental sectors, which are difficult to clean. Prolonged plaque accumulation around orthodontic brackets and bands has been demonstrated to determine a rapid shift in the bacterial flora, promoting acidogenic bacteria such as *Streptococcus mutans* and *Lactobacilli*, increasing the risk of enamel demineralization, white spot lesions and cavities [26]. Different methods have been studied to prevent cariogenic events, in particular, the efficacy of fluoride-containing products. Their efficacy is indeed strongly related to patient compliance and accuracy in domiciliary hygiene procedures [27].

Technology made it possible to add antimicrobial agents to dental resins and to cement to reduce the incidence of white spot lesions and cavities and, at the same time, maintain unchanged the adhesion properties. Companies tried to incorporate zinc oxide, chlorhexidine and fluorides to reduce the acid oral environment and decrease bacterial metabolism.

Additionally, in this case, it is hypothesized that the use of antibacterial release materials such as silver nanoparticles helps to maintain good oral hygiene during orthodontic treatment [14].

Band cements with antimicrobial characteristics (antibacterial release materials such as silver nanoparticles) have been developed to counteract the onset of white spots. These resins have mechanical properties potentially comparable to controls, and in most cases, they were found to be biocompatible. However, further studies are needed to validate the efficiency of these materials in the oral environment, considering the length of an entire orthodontic treatment [15].

## 6. Orthodontic Miniscrews

Managing the anchorage during tooth movement is a fundamental aspect of a successful orthodontic treatment. The principal aim of an orthodontic treatment is to control the anchorage and create a force system to provide the desired effect and avoid undesired movements.

**Figure 1:**
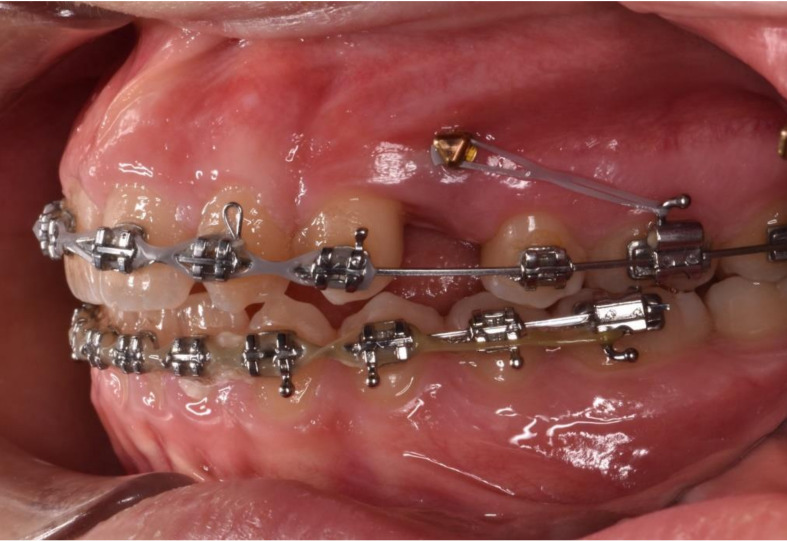
Vestibular miniscrew to enhance molar mesialization.

In different years, temporary anchorage devices (TADs) have been integrated for orthodontic purposes [28] including miniscrews, miniplates and implants [29]. They are inserted into the bone and aim to enhance orthodontic anchorage directly if they work as independent anchorage or indirectly if they support and reinforce the anchoring teeth (Figure 1).

TADs generally find their stability mechanically (cortical or bicortical stabilization) and do not require biomechanical osseointegration [29,30,31]. Currently, clinicians prefer to use miniscrews, despite the higher success rate of miniplates. The miniplate placement procedure requires an oral surgeon, and it is more invasive and expensive. Miniscrews are versatile: they are available in favorable sizes, simple to insert and remove, and affordable, and the procedure can be easily performed by a skilled orthodontist. In the literature, different insertion sites have been described, both in the maxilla and mandible. They can be placed in the vestibular bone, interradicular space [22,23], zygomatic buttress [32], symphysis [25,26] and hard palate [33,34,35].

It is therefore important to have a close intimacy between the bone and the surface of the miniscrew, as this allows better stability and greater resistance to orthodontic forces. Furthermore, inflammatory processes can affect the primary stability and determine a premature loss of the screw.

Two studies have evaluated the stability and osseointegration of miniscrews surface modified by nanotechnology: the studied surface was characterized by TiO_2_ (titanium dioxide) nanotube arrays [16,17].

The TiO_2_ nanotube arrays were loaded with RhBMP-2 (recombinant human bone morphogenetic protein-2) and ibuprofen and were compared with a control group of standard miniscrews. The effects of the drugs were evaluated in vivo: the study looked at how drug-modified miniscrews had a positive effect on tissue health.

These modified miniscrews can convey other drugs, such as antibiotic agents, aspirin and Vitamin C, to decrease inflammation at the insertion site and patient discomfort. This modification to the materials has also proved important in ensuring greater surface roughness of the aids and improving wettability compared to conventional products.

## 7. Prevention of Dental Caries and Control of Oral Biofilm

**Figure 2:**
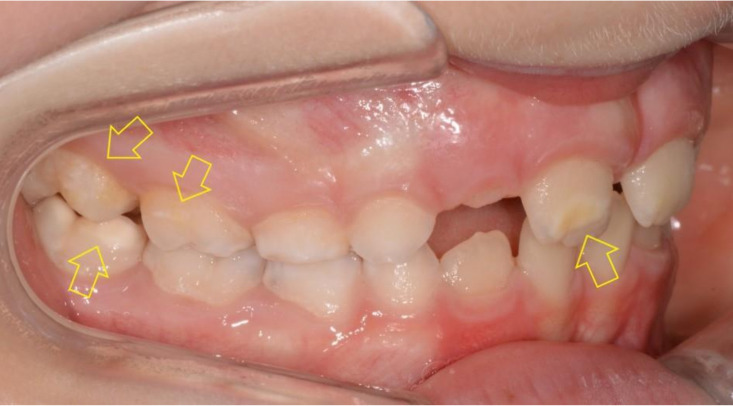
Molar incisor hypomineralization. Orthodontic treatment can affect dental tissue and lead to cavities.

One of the goals of modern dentistry is to improve prevention and reduce the need for more invasive treatments. Nanotechnology can improve the management of these aspects; in particular, it seems to address the control of bacterial biofilms and remineralization following tooth decay. In this way, it would be easier for patients to maintain good oral hygiene, especially in those young patients at high risk of dental decay (Figure 2).

White spot lesions or enamel demineralization caused by acid biofilms are very common, particularly as a consequence of orthodontic treatments, because the various orthodontic devices inevitably lead to an increase in plaque presence and retention. These lesions develop mainly in areas adjacent to orthodontic brackets [36].

To overcome the problem of caries around orthodontic brackets, antibacterial adhesives with different nanoparticles such as TiO_2_ (titanium dioxide), SiO_2_ (silicon dioxide) or SNPs (silver nanoparticles) have been tested and extensively discussed [37,38]. The incorporation of TiO_2_ nanoparticles into an orthodontic adhesive has been shown to enhance its antibacterial effects for 30 days without compromising its physical properties [38,39]. Furthermore, effective antibacterial properties have been described for SNP adhesives [37,38]. However, these appear to be of limited use in orthodontics, as they can cause enamel discoloration and cosmetic treatment results. The other approach to reducing the failure rate due to dental caries has been to modify the arches or brackets with coatings such as TiO_2_ nanoparticles doped with nitrogen [38,40] or Ag–Zr nanocomposite coatings [38,41].

The early lesions of enamel can be remineralized with dentifrices that contain nanosized calcium carbonate [1,11]. Inorganic materials such as hydroxyapatite (HA) or its derivates with zinc, fluoride, carbonate or organic compound materials present in food or beverages can be used as prophylaxis [11].

To date, some studies have evaluated the power of remineralization of toothpastes and mouthwashes based on the concepts of nanotechnology.

It was demonstrated that dentifrices containing nanosized calcium carbonate allowed early enamel remineralization [18,19]. Currently, however, the literature is controversial regarding the evidence of the efficacy of nanomaterials in improving preventive dentistry if compared to conventional ones [11].

There are, however, new studies concerning dental robots (also called dentifrobots), kinds of nanorobot that are incorporated in mouthwash or toothpaste to clean the surfaces above and below the gingival margin, and that counteract the formation of calculus, metabolizing trapped organic matter into harmless and odorless vapors; they are safely inactivated when ingested [3,11,12,13,14,15,16,17,18,20].

The perspective is that all these strategies may be an alternative to fluoride materials, especially for younger patients who are at higher risk of fluorosis [11,42].

However, some studies have shown that the presence of nanofillers in fluoride-releasing materials does not promote further benefits against carious lesion development [43]. Further studies are needed to assess the degradation and behavior of these new particles, to verify that they have no particular risks, as they are normally not in contact with the organism [11,42].

## 8. Coated Orthodontic Archwires

Orthodontic treatment consists, essentially, of sliding brackets along an archwire; however, this implies that a friction force inevitably develops between the surfaces of the two auxiliaries (the archwire and the bracket), which opposes the therapeutic movement of the teeth. To perform the dental movement, the force applied by the orthodontic appliance has to overcome this resistance. It has been measured that more than 60% of the orthodontic force applied to obtain dental movement is expected to be lost due to frictional forces [44].

The factors that mostly affect the determination of friction forces are the design of the bracket (torque), the section of the slot, the types of ligation, the section of the archwire, the inter-bracket distance and the oral functions [18,45,46].

It is easy to understand that the reduction of friction force would be advantageous to reduce treatment times and the risk of root resorption, allowing greater control of the movements and the anchorage [21,44,47].

For many years, different strategies have been sought to find solutions to reduce friction. First of all, research focused on different designs for the brackets. Secondly, different archwire alloys and surface treatments were studied.

**Figure 3:**
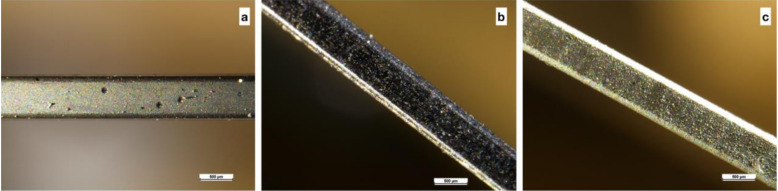
Stainless steel archwires, 0.19 × 0.25, coated with MoS_2_ (molybdenum disulfide) and W2 (tungsten disulfide).

Today, nanotechnology aims to reduce frictional forces, allowing the system to work more easily: it was suggested that the best solution is the coating of orthodontic archwires with a film incorporating nanoparticles. The best materials for achieving the goal of friction reduction are considered to be MoS_2_ (molybdenum disulfide) and W2 (tungsten disulfide) [11,21] (Figure 3).

The inorganic fullerene-like tungsten disulfide (IF-WS_2_) nanoparticles have been exploited for this purpose; it was shown that under certain reducing and sulfidizing conditions, at elevated temperatures, tungsten oxide (WO_3_) nanoparticles could form nested WS_2_ fullerene-like nanostructures, creating layers that resemble an onion or a nanotube. The layers were weakly connected through van der Waals forces only, and it is known that WS_2_ and MoS_2_ particles with layered structures (platelets) provide good lubricity [48]. The positive chemical and physical characteristics of fullerene have led engineers to develop modern inorganic fullerene-like nanoparticles, to apply these properties to new materials, even in dentistry. The attention focused in particular on chalcogenides (binary salts of S, Se or Te) of the transition metals, in particular, on the disulfides MoS_2_ and W2: they have a layer structure, similar to graphite, which is characterized by the overlapping molecular planes. These nanoparticles were firstly described by Tenne in 1992. The van der Waals forces between the layers are relatively weak, and this determines the characteristics: these nanoparticles are allowed to roll rather than slide when they are interposed between a metal and solid lubricant [11,22]. Among the main features, there is the ability to maintain an intimate contact with metal surfaces, to dissipate less energy under a force load due to elastic deformations of the nanoparticles, and to develop a lower temperature with increasing friction. The nanoparticles also develop poor reactivity owing to the bonds that characterize them, which prevent any oxidation phenomena [11].

Regarding the toxicity of these materials, some studies have demonstrated no toxic effects in rats and after dermal application [46]. Further research is necessary to demonstrate there are no toxic effects in humans for the entire length of an orthodontic treatment.

## 9. Conclusions

Today, nanotechnology has an important and more consistent role in the dental field since it has the potential to bring significant innovations and benefits. The recent positive results must be a stimulus for future research, in particular, regarding the field of orthodontics. We have explained how nanomaterials have introduced many advantages in this field, especially regarding their mechanical and antibacterial properties. The coordinated and safe management of orthodontic treatment is fundamental; the limitations of dental materials and technical procedures often refer to this goal, but science and nanotechnology have helped to partially solve some limitations, improving patient management in the clinical pathway. However, nanotechnology needs to evolve to express its maximum potential since production technical difficulties and engineering problems have not yet been completely overcome. Further studies are needed to develop fully safe and biocompatible materials [8,9,11,49,50].

## Figures and Tables

**Table 1:** Application of nanotechnology in orthodontics.

Main Fields of Application of Nanotechnology in Orthodontics
Orthodontic elastomeric ligatures [12]
Orthodontic power chains [13]
Orthodontic bands [14,15]
Orthodontic miniscrews [16,17]
Control of oral biofilm [1,3,11,18,19,20]
Coated orthodontic archwires [11,21,22]

## Data Availability

The data used to support the findings of this study are available from the corresponding author upon request.
